# Novel Techniques for Detection of *Mycobacterium bovis* Infection in a Cheetah

**DOI:** 10.3201/eid2603.191542

**Published:** 2020-03

**Authors:** Tanya J. Kerr, Rachiel Gumbo, Wynand J. Goosen, Peter Rogers, Robert D. Last, Michele A. Miller

**Affiliations:** Stellenbosch University, Cape Town, South Africa (T.J. Kerr, R. Gumbo, W.J. Goosen, M.A. Miller);; Provet Wildlife Services, Hoedspruit, South Africa (P. Rogers);; VetDiagnostix, Veterinary Pathology Services, Pietermaritzburg, South Africa (R.D. Last)

**Keywords:** cheetah, Acinonyx jubatus, novel techniques, detection, CXCL9 gene expression assay, Mycobacterium bovis, bacteria, tuberculosis and other mycobacteria, bovine tuberculosis, zoonoses

## Abstract

In South Africa, bovine tuberculosis threatens some of Africa’s most iconic wildlife species, including the cheetah (*Acinonyx jubatus*). The lack of antemortem diagnostic tests for this species strongly hinders conservation efforts. We report use of antemortem and postmortem diagnostic assays to detect *Mycobacterium bovis* infection in a cheetah.

Charismatic carnivore species such as the cheetah (*Acinonyx jubatus*) are considered a priority within conservation areas. However, the global population of cheetahs is decreasing; they are classified as vulnerable to extinction by the International Union for Conservation of Nature Red List of Threatened Species (*1*). Infectious diseases, such as bovine tuberculosis, pose a threat to wild carnivore populations, especially those that are small and fragmented (*2*). Bovine tuberculosis, caused by *Mycobacterium bovis*, affects multiple wildlife species in South Africa. Although fatal *M. bovis* infection has been described in a cheetah in Greater Kruger National Park, South Africa (*3*), reports of antemortem diagnosis in this species in South Africa have not been published. We report use of antemortem and postmortem diagnostic assays to detect *M. bovis* infection in a cheetah.

The study protocol was approved by the Stellenbosch University Research Ethics Committee (protocol no. SU-ACU-2019–10286). In January 2019, an adult female cheetah from a breeding center showed signs of chronic pneumonia. A positive test result was observed for an intradermal comparative cervical tuberculin test, based on the protocol described for African lions (*Panthera leo*) (*4*). 

Thereafter, blood was collected for additional immunologic tests, including the *CXCL9* gene expression assay (GEA) and the Dual Path Platform (DPP) Vet TB Serologic Assay (Chembio Diagnostic Systems, Inc., http://chembio.com). The use of these assays have been reported for African lions (*5**,**6*). In brief, we performed the *CXCL9* GEA by adding whole blood into QuantiFERON-TB Gold Plus tubes (QIAGEN, https://www.qiagen.com) and incubating for 24 hours at 37°C. We stabilized whole blood cell pellets by using RNALater Solution (Ambion, https://www.thermofisher.com), after which we extracted RNA by using the RiboPure Blood Kit (Ambion) and reverse transcribed this RNA by using the Quantitect Reverse Transcription Kit (QIAGEN). We performed quantitative PCR as described for lions (*5*). Using previously determined cutoff values calculated for lions, we interpreted the mycobacterial peptide-specific response for the cheetah as *CXCL9* GEA positive (2^–ΔΔCq^ = 90.09; cutoff value >27).

In addition, we tested serum by using a commercial DPP serologic assay (*6*). We detected antibodies to the antigen MBP83 (relative light units = 52.5, cutoff value >5). On the basis of these antemortem test results, the cheetah was suspected to be infected with *M. bovis* and was euthanized.

The postmortem examination and histopathologic findings showed a severe multifocal to coalescent, necrotizing, caseous, granulomatous bronchopneumonia. Lung tissues were processed for mycobacterial culture, and culture isolates were genetically speciated by using genomic regions of difference PCR and spoligotyping, as described (*7**,**8*). Using this approach, we confirmed that the cheetah was infected with *M. bovis* isolate SB0121, a strain commonly found in Greater Kruger National Park.

We also detected *M. tuberculosis* complex (MTBC) DNA in lung tissues before mycobacterial culture results were obtained by using the GeneXpert MTB/RIF Ultra Assay (Cepheid, https://www.cepheid.com) (*9*). GeneXpert PCR machines are widely available in numerous human tuberculosis clinics and laboratories across South Africa to enable rapid identification of human MTBC infections (*9*). Therefore, this tool might also be useful for MTBC diagnosis in wildlife if samples can be taken to these clinics.

Within the borders of South Africa, most cheetahs are found in fragmented populations, resulting in development of an actively managed metapopulation, which is currently composed of 60 reserves (V. van der Merwe, Endangered Wildlife Trust, pers. comm., 2019 Nov 8). The primary goal of the metapopulation is to maintain genetic diversity by translocating animals between reserves. One of the current challenges facing cheetah conservation is quarantine and restricted movement of cheetahs from premises that have known *M. bovis*–infected species present. Because movement of cheetahs from these populations requires tuberculosis testing, the lack of antemortem diagnostic tests, which can distinguish between *M. bovis*–infected and uninfected animals, strongly hinders conservation efforts.

Although tuberculosis diagnostic tests for other species rely predominantly on detection of the adaptive immune response of the host, only a few studies investigated immunoassays for wild felids (*6*). In this instance, the tuberculin skin test response was key to promoting further investigation. The decision to use the lion *CXCL9* GEA for this cheetah was based on its previously reported sensitivity of 87.5% for lions (*10*) and the assumption that various cytokine mRNA transcripts were homologous between wild felid species. This assumption was strongly supported when it appeared to be an accurate indicator of *M. bovis* infection after confirmation by mycobacterial culture. In addition, the DPP serologic assay detected a strong antibody response to *M. bovis* antigen MPB83 during antemortem screening of this cheetah. Because these findings are limited to 1 case, further investigation in a larger cohort is required before drawing conclusions about the ability of these diagnostic assays to discriminate between *M. bovis*–infected and uninfected cheetahs.

In conclusion, novel antemortem immunoassays (*CXCL9* GEA and DPP) and a postmortem PCR (GeneXpert Ultra) appear to be promising tools for detection of *M. bovis* infection in cheetahs. Tests that can accurately identify *M. bovis*–infected cheetahs are crucial to supporting conservation efforts and tuberculosis control.
